# Data imbalance in drug response prediction: multi-objective optimization approach in deep learning setting

**DOI:** 10.1093/bib/bbaf134

**Published:** 2025-04-03

**Authors:** Oleksandr Narykov, Yitan Zhu, Thomas Brettin, Yvonne A Evrard, Alexander Partin, Fangfang Xia, Maulik Shukla, Priyanka Vasanthakumari, James H Doroshow, Rick L Stevens

**Affiliations:** Computing, Environment and Life Sciences, Argonne National Laboratory, 9700 S Cass Ave, Lemont, IL 60439, United States; Computing, Environment and Life Sciences, Argonne National Laboratory, 9700 S Cass Ave, Lemont, IL 60439, United States; Computing, Environment and Life Sciences, Argonne National Laboratory, 9700 S Cass Ave, Lemont, IL 60439, United States; Leidos Biomedical Research, Frederick National Laboratory for Cancer Research, 8560 Progress Drive, Frederick, MD 21702, United States; Computing, Environment and Life Sciences, Argonne National Laboratory, 9700 S Cass Ave, Lemont, IL 60439, United States; Computing, Environment and Life Sciences, Argonne National Laboratory, 9700 S Cass Ave, Lemont, IL 60439, United States; Computing, Environment and Life Sciences, Argonne National Laboratory, 9700 S Cass Ave, Lemont, IL 60439, United States; Computing, Environment and Life Sciences, Argonne National Laboratory, 9700 S Cass Ave, Lemont, IL 60439, United States; Developmental Therapeutics Branch, National Cancer Institute, 31 Center Dr, Bethesda, MD 20892, United States; Computing, Environment and Life Sciences, Argonne National Laboratory, 9700 S Cass Ave, Lemont, IL 60439, United States; Department of Computer Science, The University of Chicago, 5730 S Ellis Ave, Chicago, IL 60637, United States

**Keywords:** drug response prediction, virtual screening, machine learning, multi-objective optimization, deep learning

## Abstract

Drug response prediction (DRP) methods tackle the complex task of associating the effectiveness of small molecules with the specific genetic makeup of the patient. Anti-cancer DRP is a particularly challenging task requiring costly experiments as underlying pathogenic mechanisms are broad and associated with multiple genomic pathways. The scientific community has exerted significant efforts to generate public drug screening datasets, giving a path to various machine learning models that attempt to reason over complex data space of small compounds and biological characteristics of tumors. However, the data depth is still lacking compared to application domains like computer vision or natural language processing domains, limiting current learning capabilities. To combat this issue and improves the generalizability of the DRP models, we are exploring strategies that explicitly address the imbalance in the DRP datasets. We reframe the problem as a multi-objective optimization across multiple drugs to maximize deep learning model performance. We implement this approach by constructing Multi-Objective Optimization Regularized by Loss Entropy loss function and plugging it into a Deep Learning model. We demonstrate the utility of proposed drug discovery methods and make suggestions for further potential application of the work to achieve desirable outcomes in the healthcare field.

## Introduction

Cancer is a widely spread genetic disease family with a common characteristic of uncontrolled cell growth and proliferation [1, 2]. This set of complex genetic disorders is highly heterogeneous and notoriously difficult to combat. Artificial intelligence technologies are being incorporated into this field to facilitate our ability to treat patients. For example, machine learning (ML) systems use doctors in processing radiological images and histopathological information.

**Figure 1:**
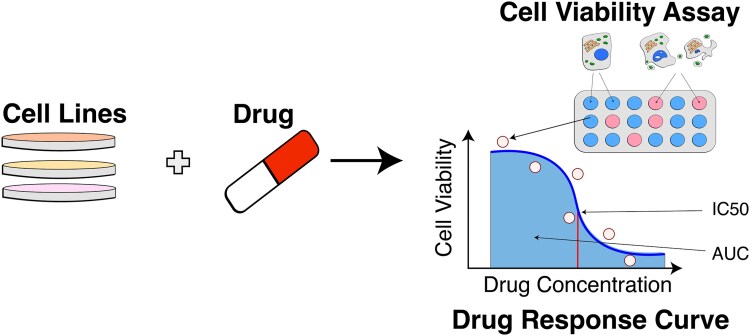
Drug response experiment. Multiple cell viability assays (a combination of membrane-permeable and membrane-impermeable fluorescent proteins that highlight tumor inhibition) are integrated into a single drug response measurement based on the hill-slope model, graphical interpretation of IC50 and AUC drug response measures [23].

Drug response prediction (DRP) is an important application of ML as it projects our estimates for the small ligand efficacy in treating cancer (Fig. 1). Designing efficient DRP models can help with real-world problems of drug repurposing, personalized medicine, and virtual drug screening by reducing the number of costly wet lab experiments required to devise novel treatment protocols or develop new drugs. However, all those settings require different approaches for assessment. Some models approach the problem in drug-specific scenarios, corresponding to personalized medicine settings, e.g., MOLI [3]. They predict drug response for a particular small molecule-based exclusively on biological information. These models cannot make inferences based on previously unseen chemical compounds. This setting severely limits the amount of information available for training and the strength of the model. To alleviate this issue and extend the application to different scenarios, most works approach DRP as a pair-input problem. This setting is called pan-drug DRP [4].

The DRP field is abundant and contains multiple models based on traditional ML approaches – Random Forest [5], AdaBoost [6], XGBoost [7], LightGBM [8], and Support Vector Machine [9]. The quantitative structure–activity relationship methods, such as kernelized Bayesian matrix factorization (KBMF), were used for the drug recommendation system [10]. Conformal prediction methods were used to assign reliability of the model prediction in DRP setting [11]. The recent trend is the extensive usage of Deep Learning (DL) models that utilize automatic feature extraction associated with multi-layer Neural Networks (NN). One of the first approaches in this direction was described by [12], who proposed a single-layer NN for predicting IC50. In recent years, the DRP field has had multiple models based on various architectures – convolutional neural networks (DeepIC50, DeepCDR, IGTD), graph neural networks (GraphDRP, GraTransDRP), attention-based models (PaccMann, CADRE, DeepTTA) [13–20].

A pair-input setting introduces known model evaluation pitfalls [21, 22], and it is important to make appropriate train/test splits to get generalizable performance estimates. So, for a drug repurposing scenario, it is natural for the model to have prior information on both biological samples and ligands. It means that the training set may include the response of the drug in question on another cell line and the response of some other drugs on a given cell line. As long as the combination of biological data and ligands is unique, including it in the test set is appropriate. However, for virtual drug screening, ensuring that the model has no prior information on the small molecule is essential. This means that if a drug appears in a test set entry, no pairs should be involved in the training set. Otherwise, we would observe information leakage and have over-optimistic results.

Personalized medicine aims to find a treatment plan best suited for a specific patient based on their biological characteristics—genetic makeup, disease history, and style of living. DRP applications in this area aim to detect drug resistivity and, for cancer, find the most efficient drug to combat tumors specific to a given patient [4, 24].

Drug discovery [25] setting is one of the most challenging applications for DRP models, as response variability between drugs is much higher than between cell line response variations [26]. It is vital for advancing drug discovery capabilities. A significant number of works in the field focus on a one-size-fits-all optimization approach when training models. In most cases, the target is to minimize the Mean Squared Error (MSE) over all pair-inputs. The performance of pan-cancer pan-drug models is commonly evaluated on a cross-validation (CV) holdout test set using performance metrics like the Pearson Correlation Coefficient (PCC) and the coefficient of determination (R2). This approach assumes the ability of ML algorithms to uncover relationships between variables automatically and hides the complexity of underlying data structures. While multiple works address the confounding factors for the prediction problem, most of them focus on information from different data modalities, e.g., copy number variations or mutation data [27–29]. However, it is necessary to understand limitation of the proposed evaluation. The drug-blind split approach in our evaluation aims to simulate virtual screening scenarios, but as noted by [30], the resulting performance metrics may not directly reflect real-world effectiveness. This limitation stems from the inherent chemical property variations across training, validation, and test datasets, which persist even when using advanced partitioning methods like scaffold splits or chemistry-based clustering. When test compounds share minimal chemical similarity with the training data, models must make out-of-distribution predictions, which remains challenging for all machine learning approaches. Therefore, for each specific screening application, training and testing splits must be carefully designed to ensure reliable predictions for the target drug classes within the screening library. The model results may vary significantly across different splits and should be compared only to the results obtained on the same split or at least similar one with the same data distribution.

**Figure 2:**
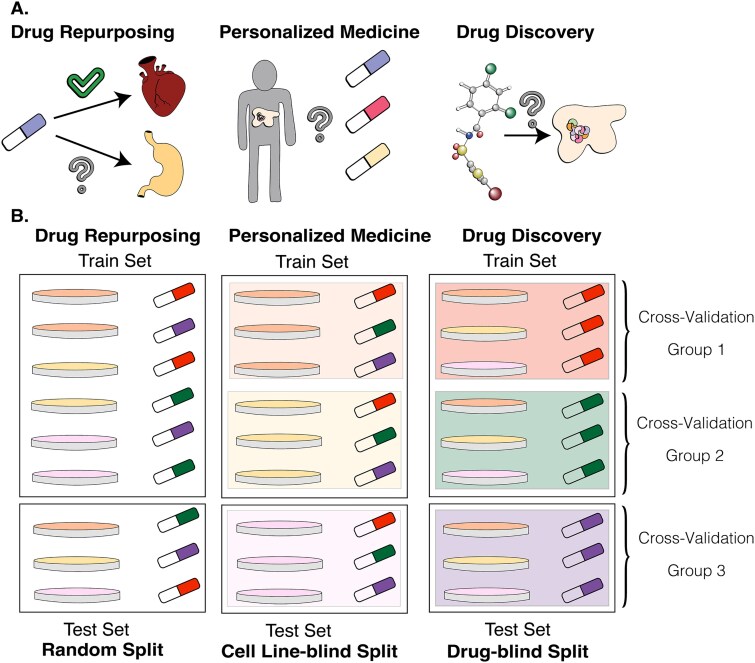
Drug response prediction application areas. A. Real-world tasks that benefit from DRP models. B. Corresponding splits of pair-input entries from drug response datasets. Each petri dish color corresponds to a unique cell line, and each color of the drug corresponds to a unique drug.

In this work, we are investigating the benefits of explicitly addressing complex substructures arising from the pair-input nature of data with a focus on improving the drug-blind response prediction performance for new drug discovery (Fig. 2). We discuss existing approaches for learning from imbalanced data and propose an outlook on drug response prediction as a multi-objective optimization (MOO) task, attempting to maximize the prediction performance over different drugs and cancers. MOO approaches usually address problems that have multiple criteria for their evaluation.

Due to the pair-input way of constructing datasets for DRP, we can approach this problem as a hybrid formulation between classification and regression tasks. While the final goal of DRP models is to predict a response value, such as the area under the dose–response curve (AUC) or the half-maximal inhibitory concentration (IC50) [31], the dataset imbalance follows conducted experiments across discrete cell lines and ligand names, which can be understood as classes. Our current work focuses on drug discovery applications, corresponding to the drug-blind split of the datasets. In addition, we are also providing an assessment for the drug repurposing task to validate proposed methods, as it is one of the standard formulations of the DRP problem [4].

## Methods

### Data

The primary data source in the DRP field is cell line experiments that measure tumor inhibition via cell viability assays. Multiple metrics characterize experimental tumor inhibition results, the most widespread being the cutoff for drug concentration that provides IC50 and AUC (Fig. 1). Those are continuous metrics, so regression models are traditionally used to estimate them.

**Figure 3:**
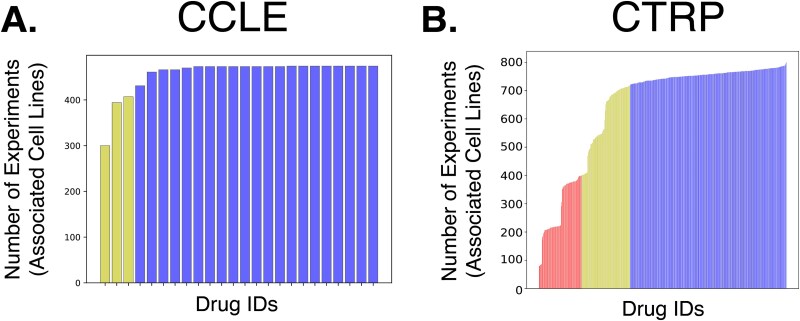
Number of experiments associated with each drug in a dataset. A. CCLE dataset. The highest number of experiments related to a single drug is 474. 12.5% of the drugs (yellow) have a number of experiments related to them that is less than 90% of the highest number. The rest of the drugs are depicted in blue. B. CTRP dataset. The highest number of experiments associated with a single drug is 799. 17.2% of the drugs (red) have a number of experiments related to them that is less than 50% of this highest number. 19.6% of the drugs (yellow) have a number of experiments between 50% and 90% of the highest number of experiments. The rest of the drugs are depicted in blue.

In this study, we use standard DRP cell line datasets – Cancer Therapeutics Response Portal (CTRP) [32] and Cancer Cell Line Encyclopedia (CCLE) [33]. CCLE dataset contains 8950 experiments based on 474 unique cell lines and 24 drugs. Data comprises RNA-Seq gene expressions, corresponding compounds, and drug response for combining those two entries. The CTRP dataset does not contain gene expression data but utilizes standard commercially available cell lines and contains a much larger number of drug response experiments - 254,566. It is based on 812 cell lines and 495 ligands. Gene expression data for biological samples came from different sources, including CCLE. We selected those datasets because they correspond to two different settings – a slight imbalance in the relatively small dataset and a significant imbalance in the large dataset (Fig. 3). Having CCLE as one of the test sets also helps us assess whether datasets with a high number of classes benefit from the proposed methodologies, regardless of the imbalance presence.

Drug-level information for both datasets comes in the form of molecular fingerprints computed via Dragon v.7.0 [34] and Simplified Molecular-Input Line-Entry System (SMILES) [35] entries obtained from the PubChem [36] and the web form of Developmental Therapeutic Program (DTP) (https://dtp.cancer.gov/). Data availability: https://zenodo.org/records/13787609 [37].

### Learning from imbalanced data

**Figure 4:**
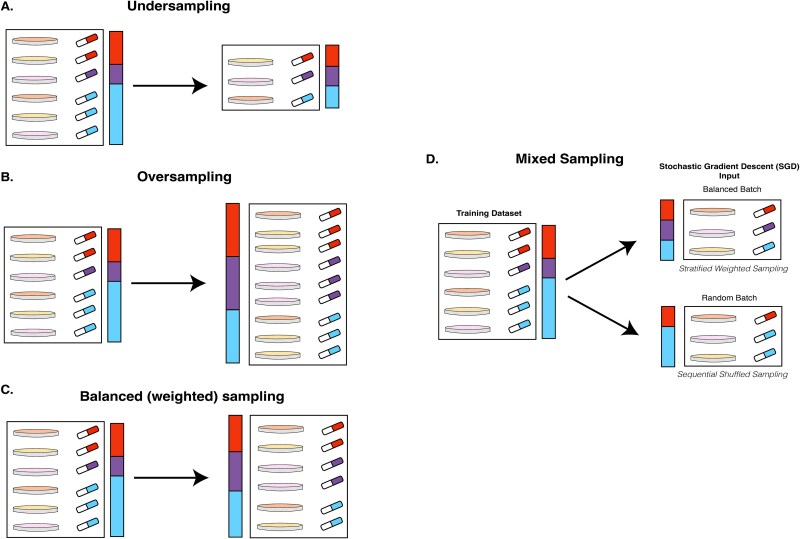
Sampling strategies for an imbalanced dataset in the context of the DRP problem. Each rectangle represents a dataset. A petri dish of a distinct color corresponds to the unique cell line. Drugs of different colors represent unique small molecules and compose distinct subgroups in data. The proportion of unique drugs is also displayed in a color bar near each dataset. In our study, we treat each drug as a class. A. Undersampling. B. Oversampling. C. Balanced, or weighted, sampling. D. Mixed sampling scheme for stochastic gradient descent-based algorithms, a variation of weighted sampling.

Data imbalance is a common problem in machine learning arising due to the limited amount of available learning data [38, 39]. This issue garnered more attention in the context of classification. Traditionally, two major approaches have been developed to handle data imbalance in the datasets: sampling (Fig. 4) and cost adaptation.

The first is focused on data preprocessing and includes various sampling techniques and synthetic data generation. It includes undersampling, oversampling, a combination of these approaches (e.g., weighted sampling) (Fig. 4), the SMOTE [40] technique, and its adaptation to the regression problem [41]. The undersampling strategy balances data by discarding excessive data in overrepresented classes. It works well when samples of the same class are similar and additional data points from that class are not crucial for making precise predictions. It is unsuitable for the DRP problem because it leads to severe data loss. Oversampling randomly draws instances from less frequent classes with replacement until the number of examples from each class is balanced. In this case, we have no data loss; however, the importance of data points from less frequent classes becomes inflated, which may introduce systematic errors to a model [39].

The second major approach focuses on the learning algorithm modifications. There is a large body of works for classification problems that attempts to introduce class weights (weighted variations of random forest [5] and SVM [9], modify the loss function, (particle-swarm optimization network [42], zSVM [43], or refine boosting approaches (AdaC1-AdaC3 [44], RareBoost [45], BABoost [46]. For regression, probability-based methods such as reframing were introduced. This approach focuses on adapting to estimated outputs depending on the context [47].

### Drug response prediction as multiple objective optimization

As we discussed earlier, the most common model evaluation is based on integral performance, e.g., ${R}^2$, PCC, concordance index, etc. In this paragraph, we are using ${R}^2$ as an example of performance measurement, and we refer to the standard evaluation of the entire hold-out portion of the dataset as ${R}_{avg}^2$. This measure compares estimates of the residual sum of squares produced by the model


(1) \begin{equation*} {R}^2=1-\frac{RSS}{TSS}=1-\frac{\sum{\left(y-\hat{y}\right)}^2}{\sum{\left(y-\overline{y}\right)}^2} \end{equation*}

where $y$ is the ground truth value, $\hat{y}$ is the model prediction, and $\overline{y}$ is the expected value of the response variable in the test dataset. Maximizing ${R}^2$ is a common target for DRP models, and can be considered a single-objective optimization problem. However, directly using ${R}^2$ for training the ML algorithms is not a common approach, as the coefficient of determination is not a convex function. It is more feasible to disregard the total sum of squares and solve the optimization problem directly for the residual sum of squares. It results in a common MSE loss function:


(2) \begin{equation*} MSE\left(f(x),y\right)=\frac{1}{n}\sum_{i=1}^n{\left(f\left({x}_i\right)-{y}_i\right)}^2 \end{equation*}

where $\mathrm{x}$ is the set of features, $n$ – total number of datapoints, $f$ – prediction model, $y$ – ground truth value, and $\hat{y}=f\left({x}_i\right)$.

**Figure 5:**
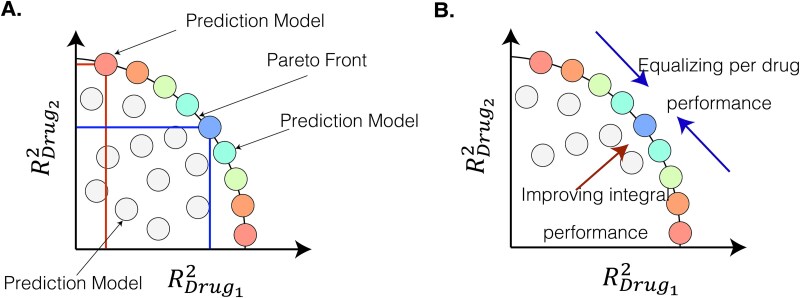
Pareto front of ML models in the space composed of individual drug performance metrics. Colored nodes indicate ML models belonging to the Pareto front, i.e., having integral performance close to the maximum known value. Grey points represent ML models that have worse integral performance.

This formulation is suitable for drug repurposing task, as it considers each unique combination of biological sample and ligand a unique standalone data sample. However, when we discuss the virtual drug screening application, we are interested in the model’s ability to reason over individual small molecules. It means that the performance of each drug in the dataset can be considered a standalone optimization problem. Let’s consider that for each drug we are attempting to maximize ${R}_{Drug_i}^2$. We can construct a data space where each coefficient of determination corresponding to *i*-th drug forms an orthonormal basis. Then each individual machine learning model can be uniquely described based on the performance it achieves for the corresponding small molecule (Fig. 5A). E.g., in two dimensional case with two drugs ${Drug}_1$ and ${Drug}_2$ vector $<{R}_{Drug_1}^2,{R}_{Drug_2}^2>$ defines the coordinates of the corresponding machine learning model. On top of individual decomposition into performance evaluations ${R}_{Drug_i}^2$ we can also associate an integral performance metric with each machine learning model. It can be either ${R}^2$ over all datapoint or an average of individual performances ${R}_{Drug_i}^2$. In this work, we choose the latter because this measure is less sensitive to the number of experiments associated with each separate drug.

We can see (Fig. 5A) that under these assumptions, multiple non-dominated data points across different axes may correspond to the machine learning models with the same integral performance but different tradeoffs between individual drugs. This is a subset of the class-composed Pareto front that is defined as all possible models with extreme performance [48]. Now, an important question is which ML model from the Pareto front is preferable. We hypothesize that selecting models closer to the center of this set (cyan and green points from Fig. 5) will result in better generalization - a capability of predicting responses to new drugs not included in the training set—as the trained models are not skewed towards some drugs and thus provide better generalizability between drugs. The reason is that such a model can provide a better association between the unique chemical characteristics of the small compounds and the biological sample’s features [37]. Figure 5B provides a graphical visualization of our objective—to maximize the integral performance of the model and to balance individual drug scores.

As in the case with the regular ${R}^2$, the current formulation does not fit to be directly used for ML algorithms training. To realize this strategy, we can define a loss function based on the MSE of individual drugs and an entropy-like regularization component. We will define loss for individual drugs as


(3) \begin{equation*} {MSE}_{d_i}= MSE\left(f\left({x}_{d_i}\right),{y}_{d_i}\right) \end{equation*}

where $\mathrm{x}$ is the set input of features derived both from cell line and drug information, ${d}_i$ is the *i*-th drug, $f(.)$ is the prediction model that produces AUC, $y$ is the ground truth values. Then, we will calculate normalized losses by applying the softmax function to individual scores and put them in the set $P$:


(4) \begin{equation*} {p}_{d_i}=\frac{\exp \left({MSE}_{d_i}\right)}{\sum_j\exp \left({MSE}_{d_j}\right)} \end{equation*}

(5) \begin{equation*}\kern-.8pc P=\left\{{p}_{d_i}:{d}_i\in D\right\} \end{equation*}

where $D$ is the set of all drugs and ${p}_{d_i}$ is normalized loss for drug ${d}_i$. As we can see, set $P$ can be treated as a probability distribution. As we want to incentivize equal loss for individual drugs, we can use a regularization based on the entropy function:


(6) \begin{equation*} H(P)=\sum_{x_j\in X}-{p}_j\log \left({p}_j\right) \end{equation*}

However, the function $H(P)$ is maximized when our desired property is achieved, and is concave. So, in order to adopt it for a loss function we will use it in form $\ln \left|D\right|-H(P)$, where $\ln \left|D\right|$ is the maximum value that discrete entropy can take for the distribution with $\left|D\right|$ entries. This transformation minimizes loss when drug-specific losses are equal and results in a convex function.

We will define the loss function over the set of features $x$, response values $y$, and classes $D$ as


(7) \begin{equation*} \boldsymbol{L}\left(\boldsymbol{x},\boldsymbol{D},\boldsymbol{y}\right)=\frac{1}{\left|D\right|}\sum_{i=1}^{\left|D\right|}{MSE}_{d_i}+a\left(\ln \left|D\right|-H(P)\right) \end{equation*}

where $a$ is a regularization coefficient, $H\left(\mathrm{X}\right)$ is the entropy of a distribution. The averaged sum of drug-specific losses maximizes integral performance, while the entropy-based regularization component promotes evening-out loss across drugs. We call this construction Multi-Objective Optimization Regularized by Loss Entropy (MOORLE) loss function.

Each individual part of the equation (7) is convex. However, due to the dependency on the model in evaluating $H(P)$ components, the resulting $\boldsymbol{L}\left(\boldsymbol{x},\boldsymbol{D},\boldsymbol{y}\right)$ may no longer be convex, even though it consists of a linear combination of convex functions. It is convex only if the model stays unchanged, which is not the case with the continuous weights update or boosting tree addition. It results in a versatile model-agnostic loss function that can be utilized both in classical ML models and deep learning settings, but it can allow more complex deep learning models to undergo more detailed tuning and make the best out of the automatic feature extraction procedure.

### Mixed sampling approach

The practical consideration regarding loss function modification proposed in 2.3 is the modern approach for NN training. Instead of updating gradients for the entire dataset in the gradient descent (GD) algorithm, continuous updates of model weights are made based on mini-batches. This approach allows to train models significantly faster and is a main feature of stochastic gradient descent (SGD) or its improvements, e.g., ADAM [49]. When we use sequential shuffled sampling that draws each element from the dataset once in random order, and is de facto a standard for deep learning frameworks like PyTorch and Keras, there is a high possibility that underrepresented drugs would have only a small influence on the objective function. See supplementary materials for the detail. We use the mixed-sampling approach as a representative data weighting method to be one of the baselines for the comparison, along with the MSE loss function.

### Machine learning algorithms

The approach described in section 2.3 is model-agnostic and can be potentially adopted for any ML algorithm; due to the latest trends in the field, we are focusing on its adaptation for Deep Learning settings. We incorporate loss function (Eq. 7) into the recent state-of-the-art model DeepTTA [20].

DeepTTA consists of three main components. One is an attention-based SMILES encoder subnetwork, and the other is a fully connected neural network (FCNN) [15]. The last part of the network concatenates encodings for drugs and biological samples and performs regression.

## Results

### Experimental setup

We analyze the effect of adopting a mixed sampling approach and entropy-regularized loss function in DeepTTA models under random split and drug-blind split CV model evaluation strategies (Fig. 2B). In a drug-blind setting, a ligand cannot appear in the training and testing sets simultaneously. It ensures that no information about a particular ligand is present in the test set. Each model run is evaluated by 10-fold cross-validation with fixed split that is shared among the studies for the same dataset and the same split type.

**Figure 6:**
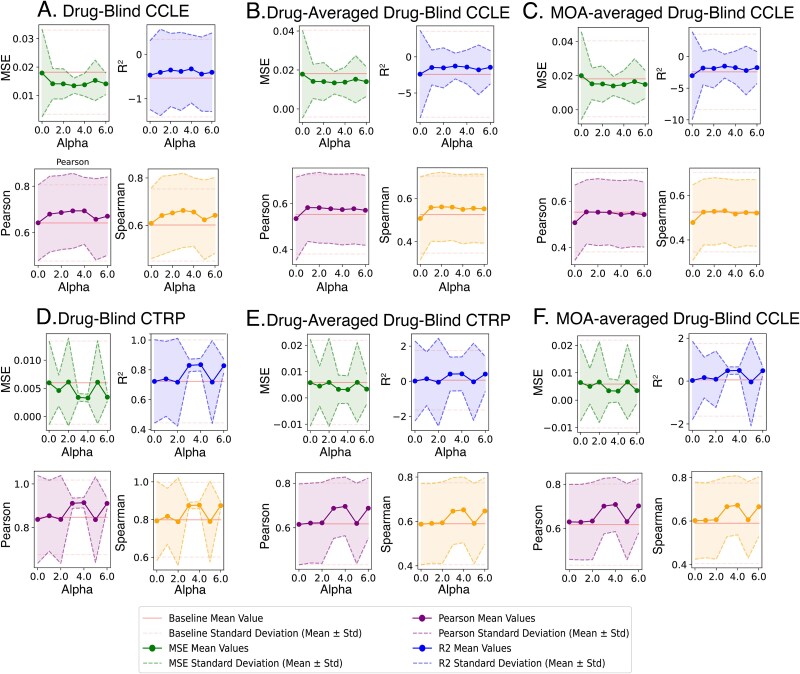
Comprehensive assessment of the regularization weight of MOORLE loss function on model performance. Each assessment includes the coefficient of determination (R^2^), mean squared error (MSE), Pearson correlation coefficient (Pearson), and spearman correlation coefficient (spearman) and their standard deviation for the varying value of regularization weight $a$ in MOORLE loss and compared to baseline performance of the model with sequential sampling and MSE loss function. A. Assessment of drug-blind cross-validation splits of the CCLE. B. Assessment of the drug-averaged performance for CCLE. C. Assessment of the MOA-averaged model performance for CCLE. D. Assessment of drug-blind cross-validation splits of the CTRP. B. Assessment of the drug-averaged performance for CTRP. C. Assessment of the MOA-averaged model performance for CTRP.

First, we perform hyperparameter tuning in the drug-blind setting of the regularization weight $a$ (Eq. 7) for the MOORLE loss function using validation splits and document its effect on model performance for the test splits (Fig. 6). We consider only drug-blind setting for these experiments due to the high computational cost. Second, we perform an ablation study to investigate the influence of sampling strategy and loss function on model performance under various conditions. We consider two sampling strategies – standard sequential and mixed sampling- proposed in Section 2.4. We also consider two loss functions – widely used MSE and multi-objective loss function with entropy regularization proposed in Section 2.3. It results in four possible combinations of the influencing factors for each model run. We use median value across 10-fold CVs for the visualization purpose due to large variations for R^2^ values. The same information is presented via a boxplot that reflects the overall data distribution and supports our conclusions in Supplementary Fig. 1.

To estimate the statistical significance of the effect that proposed strategies have on the drugs we apply two-way repeated measurements ANOVA [50] algorithm from *pengouin* Python package. For random split and drug-blind settings, results for each CV iteration are considered repeated measurement; for drug-averaged drug-blind settings, each individual drug plays this role. Greenhouse–Geisser corrected p-values [51] are reported in the findings.

### Insights from hyperparameter tuning for the drug-blind setting

We evaluated the model’s performance with the regularization parameter $a$ ranging from 0 to 6 using 10-fold cross-validation on CCLE and CTRP datasets. The data includes averaged performance metrics for bulk data from each cross-validation split (Fig. 6A, D), and averaged model performance for each individual drug (Fig. 6B, E) and drug’s mechanism of action (MOA) (Fig. 6C, F) on the test splits. In each case, we estimate the average of the metric and standard deviation, comparing those to the baseline performance of the model with the MSE loss (denoted in red color on Fig. 6). In addition to drug averaging, we analyze MOA grouping to investigate regularization effects on larger meaningful drug subgroups.

We find out that, generally, the effects of the regularization are positive both for CCLE and CTRP datasets; however, due to the subgroups-based nature of the regularization, its weight should be explored and adjusted based on a specific dataset because they may be non-monotonous. Both the CCLE and CTRP weight interval of [3.0,4.0] produce optimal values, improving average performance and reducing standard deviation. Effects on MSE and R^2^ mirror each other, which was not an obvious conclusion due to the unbound left side of the R^2^ metric. It means that the addition of the MOORLE regularization component can robustly improve its average ability to explain variations for each individual drug. The variability in the results observed for CTRP for the regularization coefficient $a$ of the value of 5.0 highlights the non-linear nature of the proposed loss function and emphasizes the need for fine-tuning it on a validation set. The reduced variability for CCLE is likely due to the smaller dataset size and more balanced drug representation.

### Random split evaluation (drug repurposing)

**Figure 7:**
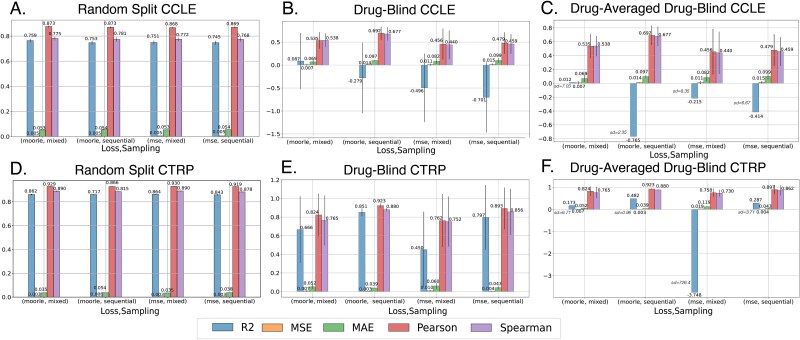
Ablation study on CCLE and CTRP datasets. Performance metrics including coefficient of determination (R^2^), mean squared error (MSE), mean absolute error (MAE), Pearson correlation coefficient (Pearson), and spearman correlation coefficient (spearman) are recorded for each combination of factors – Sampling strategy and loss function. Each bar represent median of a 10-fold CV run results. Black bar represents one standard deviation. Sampling strategies consist of sequential random sampling (denoted ‘sequential’ in the figure) and hybrid strategy introduced in 2.4 (‘mixed’ in the figure). Loss functions are represented by MSE (‘MSE’ in the figure) and MOORLE - multi-objective loss function regularized by loss entropy (‘MOOLRE’ in the figure). A. Random split evaluation strategy, CCLE dataset. B. Drug-blind split evaluation strategy, CCLE dataset. C. Drugwise evaluation under drug-blind split, CCLE dataset. D. Random split evaluation strategy, CTRP dataset. E. Drug-blind split setting, CTRP dataset. F. Drugwise evaluation under drug-blind split, CTRP dataset.

The random split was mainly introduced as a baseline setting to observe model behavior under the standard for the field experiment setup. We expected that the proposed change in the objective would not significantly influence this scenario, as the model has abundant information on the related ligands, unless we encounter a high imbalance of drug representations in batches. Indeed, as we can see, for CCLE (Fig. 7A), with R^2^ varying from 0.745 to 0.759 and MSE staying around 0.005, while two-way repeated measurements ANOVA did not detect statistically significant effects of loss function or sampling strategy on the performance. The situation with CTRP (Fig. 7B) is very similar, except for the combination of multi-objective function and sequential sampling strategy. We hypothesize that due to the completely random proportion of classes (drugs) in training batches, the model has difficulties learning the drug repurposing objective. The comprehensive list of scores for Fig. 7 is in Supplementary Table 1.

### Drug-blind evaluation (virtual screening, bulk assessment)

As described in Fig. 2, drug-blind evaluation corresponds to the virtual screening problem, where we assess the ligand’s performance previously not seen by the model. In this scenario, performance metrics are calculated for the entire hold-out portion of the cross-validation set.

As the drug-blind setup is much more challenging for the ML models, we see a sharp drop in the performance. For the CCLE dataset, the best-performing combination is MOORLE loss function with mixed sampling with MAE = 0.069, while the rest of the values vary from 0.082 to 0.099 for MOORLE with sequential sampling, MSE with mixed sampling, and MSE with sequential sampling. For the CTRP dataset, we see a combination of sequential sampling and MOORLE loss function having a slight edge over the other variations with MAE = 0.039 against 0.052, 0.060, 0.043 for MOORLE with mixed sampling, MSE with mixed sampling, and MSE with sequential sampling for CTRP dataset.

For the CCLE dataset, the two-way repeated measurements ANOVA test highlights no statistical significance of the factors on performance measures for R^2^ and MSE. For CTRP, only the sampling strategy was above the significance threshold ($\mathrm{p}-\mathrm{value}=4.67\cdot{10}^{-3}$).

### Drugwise scoring under drug-blind split (virtual screening, drug-specific assessment)

Most works that discuss drug-blind evaluation perform bulk assessment, as described in 3.3. However, to be completely thorough with our assessment, we first attempt to calculate the corresponding metric for each drug individually and then average the results. As we can see from Fig. 7E and F, the only measure significantly impacted by this procedure change is R^2^. In the case of a drug-wise assessment, this measure reflects a goodness of fit of the trained model for each given drug. However, even when the mean values of the majority of the performance metrics stay the same, taking a look at the problem from a drug-by-drug perspective allows us to better reason over the influence that changes in ML model have on the performance. As MAE score remains very close to the previous scenario, we are making comparisons based on R^2^ in this section.

For the CCLE dataset, the best-performing combination is MOORLE loss function with mixed sampling with R^2^ = 0.12, while the rest of the values are −0.765, −0.215, −0.414 for MOORLE with sequential sampling, MSE with mixed sampling, and MSE with sequential sampling for CCLE dataset. For the CTRP dataset, we see a combination of sequential sampling and MOORLE loss function having a significant edge over the other variations with R^2^ = 0.482 against 0.173, −3.748, 0.287 for MOORLE with mixed sampling, MSE with mixed sampling, and MSE with sequential sampling.

**Figure 8:**
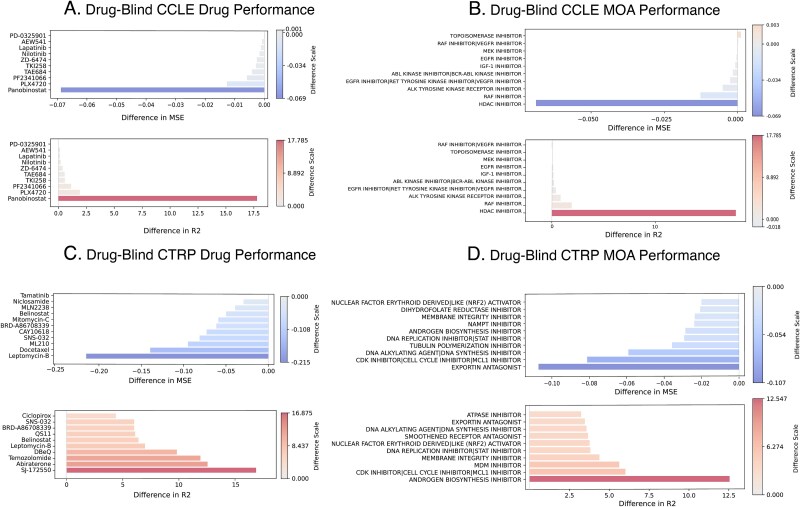
Top-10 drugs’ and MOAs’ performance improvements. Each panel visualizes the performance improvement for a specific drug or MoA, with blue bars indicating positive improvement and red bars indicating negative change. The horizontal axis represents the magnitude of improvement and the vertical axis corresponds to the individual item, a drug or MOA. We can see that improvement patterns across prediction groupings based on individual drug and molecular MOA are on a comparable scale. A. Top-10 drug performance gains on drug-blind cross-validation splits of the CCLE. B. The drug-averaged performance for CCLE. C. The MOA-averaged model performance for CCLE. D. Drug-blind cross-validation splits performance of the CTRP.

For the drug-averaged evaluation on CTRP dataset (Fig. 7F), two-way repeated measurements ANOVA test corroborated the statistically significant effect of both sampling strategy and loss, adding both a new sampling strategy ($\mathrm{p}-\mathrm{value}=4.41\cdot{10}^{-2}$), and the loss function ($\mathrm{p}-\mathrm{value}=3.39\cdot{10}^{-2}$) on R^2^ value, as well as their combination ($\mathrm{p}-\mathrm{value}=2.33\cdot{10}^{-2}$) and the outstanding impact of loss function on MSE score: sampling ($\mathrm{p}-\mathrm{value}=2.88\cdot{10}^{-280}$), loss function ($\mathrm{p}-\mathrm{value}=1.26\cdot{10}^{-123}$), and their combination ($\mathrm{p}-\mathrm{value}=1.27\cdot{10}^{-37}$). For CCLE, no factors were found statistically significant (Fig. 7C). The list of scores for the top-performing drugs are in Supplementary Table 3. The drugs and MOAs with the best scores gains are listed in Fig. 8.

We also conducted experiments on a 5-fold drug-blind cross-validation split using popular DRP methods – LightGBM [8], GraphDRP [16], and fully-connected neural network (FCNN) [12] in addition to DeepTTC with a regularization coefficient $\alpha =4$ for the DeepTTC, GraphDRP, and FCNN models, and $\alpha =0.25$ for LightGBM (Table 1). We report the average value from the folds and the standard deviation. We generated a training dataset for each model individually to accommodate for differences in the preprocessing steps, so the performance metrics should not be compared across models but only used to assess the effect of the loss function. The difference in the number of input features in the models contributes to the different outcomes for the CCLE dataset. The results showcase the utility of the MOORLE loss function for DeepTTC and GraphDRP, while its utility for boosting and FCNN remains inconclusive. This behavior may be attributed to the fact that DeepTTC and GraphDRP utilize flexible representation of the small compounds using explainable substructure fingerprints [52] and graphical representation correspondingly, while LightGBM and FCNN rely on Dragon 7 descriptors.

**Table 1:** Performance of the community models on 5-fold drug-blind cross-validation.

Dataset	CCLE		CTRP	
Performance Metric	R^2^		R^2^	
Loss function	MSE Loss	MOORLE	MSE Loss	MOORLE
Model				
DeepTTC	−0.48 $\pm$ 1.034	0.41 $\pm$ 0.170	0.79 $\pm$ 0.059	0.84 $\pm$ 0.021
LightGBM	−0.92 $\pm$ 0.121	−0.39 $\pm$ 0.107	0.71 $\pm$ 0.073	0.66 $\pm$ 0.094
GraphDRP	0.64 $\pm$ 0.026	0.66 $\pm$ 0.019	0.72 $\pm$ 0.011	0.73 $\pm$ 0.009
FCNN	0.26 $\pm$ 0.078	0.25 $\pm$ 0.065	0.83 $\pm$ 0.006	0.82 $\pm$ 0.004

## Discussion

Current drug response prediction approaches implicitly rely on the ability of deep learning algorithms to find a true relationship between features and response values while simultaneously correcting for data imbalance. However, as the amount of available data for this biomedical problem is limited, we investigated potential ways to improve predictive value by explicitly addressing the data imbalance problem.

Our contributions include the proposal of the novel model-agnostic multi-objective loss function with entropy-based regularization, and can be utilized both with classical ML algorithms and in Deep Learning. Classes, or domains $\boldsymbol{D}$, in the MOORLE loss function $\boldsymbol{L}\left(\boldsymbol{x},\boldsymbol{D},\boldsymbol{y}\right)$ are interchangeable and can guide model to be aware of other imbalances encoded in data, e.g., sex or age, that regularly appear in biomedical datasets. The proposed approach can be used to promote equitable outcomes in healthcare models. We conduct comprehensive assessments on the effects of regularization weight for drug response prediction problems, compare outcomes to regular MSE loss and weighted sampling, and study a combination of the latter with new loss function via ablation analysis. The MOORLE loss improves performance metrics and reduces standard deviation on all datasets. On top of it, its main feature is the aggregation method for data subgroup losses, which makes it extremely versatile, with the ability to switch out basic MSE loss for any other non-negative loss function. In case when we want our model to prioritize a specific drug group (e.g., ligands for the single protein target) it is possible to adjust individual loss weights to skew performance towards drugs of interest.

One of the drawbacks of the proposed methodology is the dependency on the regularization coefficient. It should be derived in the inner cross-validation loop to achieve the best possible performance. However, nested cross-validation for the proposed datasets is exceptionally computationally demanding, increasing the runtime by an order of magnitude. Our future direction is to perform a large-scale analysis of the community models with multiple loss functions and extensive hyperparameter optimization. Though our evaluation using drug-blind splits is intended to approximate a virtual screening problem, the performance evaluation may not be directly translatable to real-world applications, as cautioned by [30]. This happens due to variability in the chemical properties of the compounds that end up in training, validation, and test sets, even in the alternative grouped cross-validation settings like scaffold split and chemistry-based clustering. In case there is no similarity between chemical compounds in training and testing data, the model will be making out-of-distribution predictions, a challenging task for every ML approach. Thus, training and testing splits should be carefully tuned for each real-world application to ensure robust results on the drug class of interest in a screening library. The same considerations apply to the new domain applications, such as drug-target prediction [53] or precision medicine.

The mixed sampling strategy positively impacted the small, better-balanced CCLE dataset and was mostly detrimental to CTRP. It is possible that because of the large number of drugs in the latter, each balanced batch did not contain enough representative samples for the corresponding drug (class). Further adjustments are needed to control the number of classes sampled in a single batch. At the same time, the multi-objective loss function with entropy regularization was the primary influence for the CTRP dataset.

**Key Points**

- Data imbalance in dataset subgroups results in systematic errors in machine learning model
- Traditional loss functions like Mean Squared Error do not distinguish between subgroups
- Multi-objective optimization (MOO) gives researchers control over dataset subgroups’ treatment
- The proposed model-agnostic loss function (Multi-Objective Optimization Regularized by Loss Entropy) incorporates MOO principles and significantly improves the generalization capability of a drug response prediction artificial intelligence (AI) model in drug discovery setting
- Incorporating MOO principles in AI models can help achieve more equitable outcomes in healthcare

## Supplementary Material

MOORLE_BiB_S_Figure1_bbaf134

Narykov-MOORLE-Briefings-in-bioinformatics-Supplementary-Revision1_bbaf134

## Date availability

The full dataset is shared via zenodo: https://zenodo.org/records/13787609. The source code is available on GitHub: https://github.com/AlexandrNP/MOORLE.
