# Supplementary figures and images for: Data imbalance in drug response prediction: multi-objective optimization approach in deep learning setting
Source: Brief Bioinform. 2025 Apr 3;26(2):bbaf134. doi: 10.1093/bib/bbaf134 (PMC11966611; doi:10.1093/bib/bbaf134)

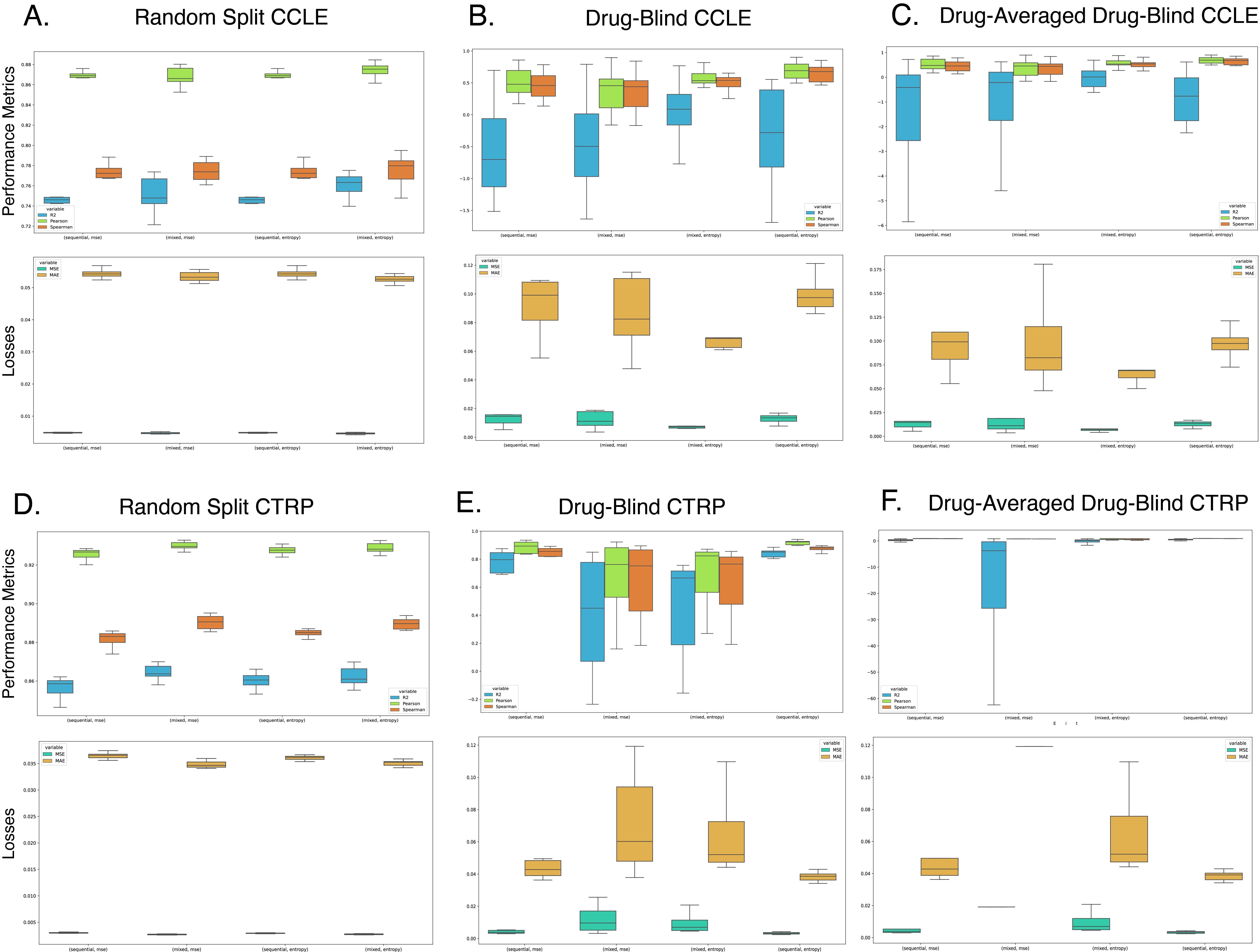

Supplement: MOORLE_BiB_S_Figure1_bbaf134 [file moorle_bib_s_figure1_bbaf134.jpeg]
