# Supplementary material for: Data imbalance in drug response prediction: multi-objective optimization approach in deep learning setting
Source: Brief Bioinform. 2025 Apr 3;26(2):bbaf134. doi: 10.1093/bib/bbaf134 (PMC11966611; doi:10.1093/bib/bbaf134)
Supplement: Narykov-MOORLE-Briefings-in-bioinformatics-Supplementary-Revision1_bbaf134 [file narykov-moorle-briefings-in-bioinformatics-supplementary-revision1_bbaf134.docx]

Data Imbalance in Drug Response Prediction – Multi-Objective Optimization Approach in Deep Learning Setting. Supplemementary Material

Oleksandr Narykov^1,*^, Yitan Zhu^1^, Thomas Brettin^1^, Yvonne A. Evrard^2^, Alexander Partin^1^, Fangfang Xia^1^, Maulik Shukla^1^, Priyanka Vasanthakumari^1^, James H. Doroshow^3^ and Rick L. Stevens^1,4^

^1^Computing, Environment and Life Sciences, Argonne National Laboratory, Lemont, IL 60439, USA, ^2^Leidos Biomedical Research, Frederick National Laboratory for Cancer Research, Frederick, MD 21702, USA, ^3^Developmental Therapeutics Branch, National Cancer Institute, Bethesda, MD 20892, USA, ^4^Department of Computer Science, The University of Chicago, Chicago, IL 60637, USA

**Supplementary Figure 1. Ablation study results visualized via boxplots. Outliers are hidden.**


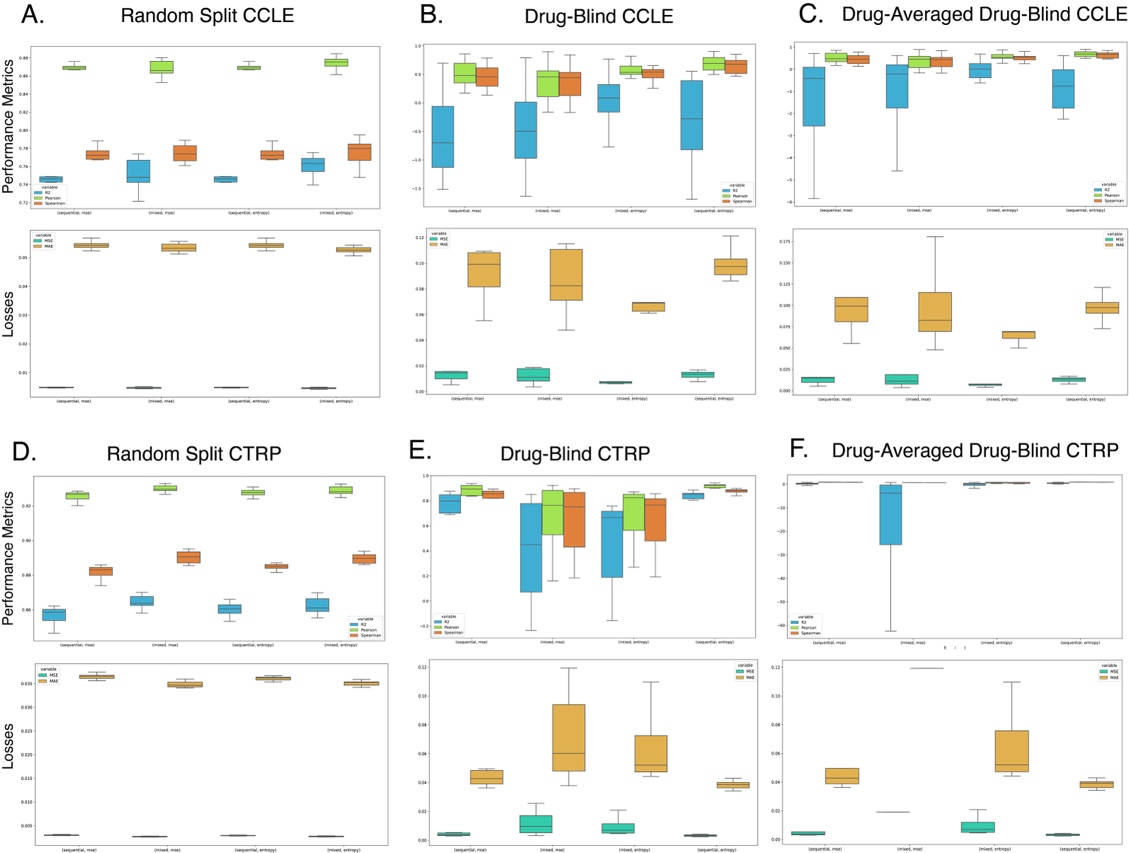


**Supplementary Table 1. Results of the ablation study.** This table contains numerical values for the data presented in Fig. 6 of the main manuscript.

| Evaluation Strategy | Dataset | Sampling | Loss | R2 | MSE | MAE | Pearson | Spearman |
| --- | --- | --- | --- | --- | --- | --- | --- | --- |
| Random Split | CCLE | mixed | entropy | 0.75940277 | 0.00461279 | 0.05263891 | 0.8734642 | 0.77524132 |
|  |  | mixed | mse | 0.75138341 | 0.00476475 | 0.0534148 | 0.8680828 | 0.7719337 |
|  |  | sequential | entropy | 0.7450857 | 0.00488475 | 0.0542805 | 0.86902626 | 0.76771261 |
|  |  | sequential | mse | 0.7450857 | 0.00488475 | 0.0542805 | 0.86902626 | 0.76771261 |
|  | CTRP | mixed | entropy | 0.86211982 | 0.00274641 | 0.0351018 | 0.92876387 | 0.88960698 |
|  |  | mixed | mse | 0.86445888 | 0.00270026 | 0.03482292 | 0.92995885 | 0.89025703 |
|  |  | sequential | entropy | 0.86032674 | 0.00293068 | 0.03607458 | 0.92770237 | 0.88482045 |
|  |  | sequential | mse | 0.85611219 | 0.00301883 | 0.03665117 | 0.92535883 | 0.88088433 |
| Drug Blind | CCLE | mixed | entropy | -0.0087928 | 0.01653774 | 0.08976977 | 0.57141596 | 0.52809053 |
|  |  | mixed | mse | -0.4392929 | 0.02157441 | 0.10556992 | 0.37814165 | 0.35358589 |
|  |  | sequential | entropy | -0.3262376 | 0.01376402 | 0.09789331 | 0.69382207 | 0.65635946 |
|  |  | sequential | mse | -0.5506573 | 0.02098103 | 0.10909531 | 0.51032025 | 0.4646368 |
|  | CTRP | mixed | entropy | 0.46758707 | 0.01003174 | 0.06391222 | 0.69628324 | 0.63742054 |
|  |  | mixed | mse | 0.39409254 | 0.01143625 | 0.06894375 | 0.65649774 | 0.65095396 |
|  |  | sequential | entropy | 0.83447079 | 0.00328879 | 0.0382537 | 0.9140203 | 0.87636624 |
|  |  | sequential | mse | 0.64215811 | 0.00624659 | 0.05181633 | 0.793043 | 0.74238959 |
| Drug Blind Drugwise Results | CCLE | mixed | entropy | -2.123909 | 0.01653774 | 0.08976977 | 0.57141596 | 0.52809053 |
|  |  | mixed | mse | -3.0311056 | 0.02157441 | 0.10556992 | 0.37814165 | 0.35358589 |
|  |  | sequential | entropy | -1.4109016 | 0.01376402 | 0.09789331 | 0.69382207 | 0.65635946 |
|  |  | sequential | mse | -2.8675557 | 0.02098103 | 0.10909531 | 0.51032025 | 0.4646368 |
|  | CTRP | mixed | entropy | -1.2516117 | 0.01000449 | 0.06381576 | 0.6974615 | 0.63872357 |
|  |  | mixed | mse | -726.95917 | 0.01883973 | 0.11174181 | 0.69299434 | 0.68076701 |
|  |  | sequential | entropy | 0.42152583 | 0.00329176 | 0.03826362 | 0.91399037 | 0.87632033 |
|  |  | sequential | mse | -0.5189049 | 0.00622447 | 0.05172703 | 0.79404115 | 0.74350025 |

**Supplementary Table 2**

**Supplementary Table 2. ML algorithm parameters**

| use_lincs = False |
| --- |
| target_id = 'AUC' |
| transformer_emb_size_drug = 128 |
| dropout = 0.2 |
| transformer_n_layer_drug = 8 |
| transformer_intermediate_size_drug = 512 |
| transformer_num_attention_heads_drug = 8 |
| transformer_attention_probs_dropout = 0.1 |
| transformer_hidden_dropout_rate = 0.1 |
| learning_rate = 1e-4 |
| optimizer=’adam’ |
| batch_size = 256 |
| epochs = 100 |
| input_dim_drug_classifier = 128 |
| input_dim_gene_classifier = 256 |
| input_dim_binding_classifier = 64 |

**Supplementary Table 3. Top-10 drugs performances for CCLE and CTRP.**

| CCLE |  |  |  | |  | |  | |  | |  | |
| --- | --- | --- | --- | --- | --- | --- | --- | --- | --- | --- | --- | --- |
| Drug | Sampling | R2 | | MSE | | MAE | | Pearson | | Spearman | |  |
| Topotecan | mixed | 0.47375 | | 0.04755 | | 0.16989 | | 0.27417 | | 0.25431 | |  |
| AZD6244 | mixed | 0.40098 | | 0.00724 | | 0.06933 | | 0.66618 | | 0.57165 | |  |
| AZD0530 | mixed | 0.31140 | | 0.00692 | | 0.06614 | | 0.50660 | | 0.42792 | |  |
| Lapatinib | mixed | 0.23947 | | 0.00616 | | 0.06144 | | 0.49825 | | 0.47264 | |  |
| Nilotinib | mixed | 0.12248 | | 0.00786 | | 0.06943 | | 0.81882 | | 0.65276 | |  |
| ZD-6474 | mixed | 0.06044 | | 0.00611 | | 0.06107 | | 0.42572 | | 0.42498 | |  |
| Erlotinib | mixed | 0.02424 | | 0.00754 | | 0.06851 | | 0.56406 | | 0.50419 | |  |
| RAF265 | mixed | -0.07558 | | 0.00719 | | 0.06940 | | 0.58288 | | 0.58774 | |  |
| PF2341066 | mixed | -0.17658 | | 0.00611 | | 0.06107 | | 0.42572 | | 0.42498 | |  |
| L-685458 | mixed | -0.34658 | | 0.00692 | | 0.06614 | | 0.50660 | | 0.42792 | |  |
| CTRP |  |  | |  | |  | |  | |  | |  |
| Drug | Sampling | R2 | | MSE | | MAE | | Pearson | | Spearman | |  |
| Docetaxel | sequential | 0.85050 | | 0.00355 | | 0.04024 | | 0.92786 | | 0.89638 | |  |
| KX2-391 | sequential | 0.84661 | | 0.00374 | | 0.04119 | | 0.92107 | | 0.86668 | |  |
| CHM-1 | sequential | 0.84067 | | 0.00340 | | 0.03912 | | 0.90344 | | 0.85877 | |  |
| Parbendazole | sequential | 0.83977 | | 0.00309 | | 0.03814 | | 0.94139 | | 0.88857 | |  |
| YK-4-279 | sequential | 0.83900 | | 0.00423 | | 0.04292 | | 0.93474 | | 0.87998 | |  |
| FQI-1 | sequential | 0.83676 | | 0.00423 | | 0.04292 | | 0.93474 | | 0.87998 | |  |
| Tivantinib | sequential | 0.83204 | | 0.00374 | | 0.04119 | | 0.92107 | | 0.86668 | |  |
| BRD-K70511574 | sequential | entropy | 0.83106 | | 0.00427 | | 0.03946 | | 0.86005 | | 0.83911 | |
| Triazolothiadiazine | sequential | entropy | 0.81772 | | 0.00238 | | 0.03434 | | 0.92542 | | 0.89702 | |
| GSK461364 | sequential | entropy | 0.81463 | | 0.00309 | | 0.03688 | | 0.90371 | | 0.88007 | |

**Mixed sampling**

Depending on the number of classes and present imbalance, oversampling may significantly inflate the size of the dataset. Balanced (weighted) sampling can be seen as a combination of the previous two approaches. Each data point is assigned a weight inversely proportional to the number of corresponding class instances in the dataset.

The SMOTE technique is based on *k* nearest neighbors and generates synthetic examples as a weighted average between a selected point and each of its neighbors (closest data points) from the same class [36]. The algorithm was adopted for regression; however, it heavily relies on the assumption of linearity between features and response value, as well as the convexity of the clusters formed by different classes, which are not observed in DRP problem data. It is also known that in high-dimensional space, SMOTE tends to be severely biased towards underrepresented classes [34]. Because of these points, we do not further consider SMOTE in our work.

Due to performance considerations stated in the paper, we explored different sampling strategies described in 2.2. Undersampling, as expected, led to unsatisfactory performance for both CCLE and CTRP datasets, as most of the data was discarded. Oversampling was suitable for smaller-sized CCLE, but in the case of CTRP, this approach inflated the training dataset 20 times its original size and was not computationally feasible. Weighted sampling allowed us to control the size of the training dataset manually. However, maintaining the dataset size two to four times the original training set resulted in data loss and performance deterioration.

This led us to develop a hybrid sampling strategy. Based on the common training dataset, we are deriving two sets of batches – one is the balanced batch based on weighted sampling, and another is a batch composed via sequential shuffle split (Fig.6). This mixed strategy inflates the size of the original training set twice and allows us to present model all samples from training dataset while ensuring regular update of cost function based on all present groups (classes) in the dataset. In practice, performing weighted sampling for each epoch significantly increases training time. To offset the cost of performing it, we cache obtained batches and store them for 10 epochs (including current), shuffling batch order before each new epoch. Using this heuristic, we reduce the number of times we have to perform weighted sampling.

**GenAI Usage Statement**

The authors used Grammarly for spelling and grammar check.
